# Right-lateralised lane keeping in young and older British drivers

**DOI:** 10.1371/journal.pone.0203549

**Published:** 2018-09-06

**Authors:** Gemma Learmonth, Gesine Märker, Natasha McBride, Pernilla Pellinen, Monika Harvey

**Affiliations:** 1 Centre for Cognitive Neuroimaging, Institute of Neuroscience and Psychology, University of Glasgow, Glasgow, United Kingdom; 2 School of Psychology, University of Glasgow, Glasgow, United Kingdom; Universite de Bordeaux, FRANCE

## Abstract

Young adults demonstrate a small, but consistent, asymmetry of spatial attention favouring the left side of space (“pseudoneglect”) in laboratory-based tests of perception. Conversely, in more naturalistic environments, behavioural errors towards the *right* side of space are often observed. In the older population, spatial attention asymmetries are generally diminished, or even reversed to favour the right side of space, but much of this evidence has been gained from lab-based and/or psychophysical testing. In this study we assessed whether spatial biases can be elicited during a simulated driving task, and secondly whether these biases also shift with age, in line with standard lab-based measures. Data from 77 right-handed adults with full UK driving licences (i.e. prior experience of left-lane driving) were analysed: 38 young (mean age = 21.53) and 39 older adults (mean age = 70.38). Each participant undertook 3 tests of visuospatial attention: the landmark task, line bisection task, and a simulated lane-keeping task. We found leftward biases in young adults for the landmark and line bisection tasks, indicative of pseudoneglect, and a mean lane position towards the right of centre. In young adults the leftward landmark task biases were negatively correlated with rightward lane-keeping biases, hinting that a common property of the spatial attention networks may have influenced both tasks. As predicted, older adults showed no group-level spatial asymmetry on the landmark nor the line bisection task, but they maintained a mean rightward lane position, similar to young adults. The 3 tasks were not inter-correlated in the older group. These results suggest that spatial biases in older adults may be elicited more effectively in experiments involving complex behaviour rather than abstract, lab-based measures. More broadly, these results confirm that lateral biases of spatial attention are linked to driving behaviour, and this could prove informative in the development of future vehicle safety and driving technology.

## 1. Introduction

Young adults exhibit a small, but consistent, spatial attention bias towards the left side of space (“*pseudoneglect”* [1]), probably as a result of right cerebral hemisphere dominance for spatial attention. Spatial asymmetries have been elicited across a range of laboratory-based tasks, involving visual judgements of size (the landmark and line bisection tasks [2]), luminance (greyscales task [3]), spatial frequency (gratingscales task [4]), emotions (chimeric face task [5]), and tasks including non-visual set-ups (the mental number line [6] and tactile line bisection tasks [1]). We have previously demonstrated consistent within-task spatial biases when 5 tasks were tested on two separate days, yet failed to find between-task consistency in young adults [7] (see also [3] and [8]). Relative to young adults, healthy older adults typically exhibit a group-level rightward shift of spatial bias, with either no spatial asymmetry, or a mild preference for the right hemispace ([9–20] but see [21,22] for maintained leftward biases in older age). This behavioural shift may represent diminished right hemisphere control of spatial attention in older adults [10].

Although pseudoneglect is most commonly assessed using computerised and/or laboratory-based tasks, spatial attention asymmetries can also be elicited in more ecologically valid contexts. Such measures are important, due to the potentially negative consequences of misjudging our environment, for example colliding with objects. This could be particularly consequential for older adults, because impaired lateral spatial processing has been associated with an increased risk of falls [14,16]. In contrast to lab-based measures, these ‘real-world’ tasks have, by-and-large, identified systematic behavioural errors and preferences towards the *right* side of space. For instance, people tend to pass through doorways to the right of true centre [23], and doorframe collisions are more likely to occur on the right side of the body than the left [24]. Furthermore, right-sided bumping also occurs when navigating doorways using electric wheelchairs, scooters and remote-controlled cars [25–27]). It is possible that the dimensions of left hemispace are perceptually overestimated and compensated for by shifting behaviourally rightward as a result. Though intriguingly, this behaviour has also been demonstrated in situations that do not appear to involve obstacle avoidance or navigation. For instance, people preferentially select seats towards the right side of an aeroplane rather than the left ([28] although see [29]), and the right side of theatre halls [29–33], however this may be dependent on the expected cognitive demands of the situation (see [34] for discussion of a leftward seating preference in classrooms).

There is good evidence that these biases are a product of asymmetrical cortical activity between the left and right cerebral hemispheres, given that behavioural biases can be modulated by activating one hemisphere unilaterally [24]. Importantly, such behavioural errors are often inversely correlated with standard lab-based tests of perceptual spatial bias, suggesting that a common neural mechanism underpins both lab-based and naturalistic tasks (e.g. [35,36]). It is nevertheless important to note that the literature does not reach full consensus: Nicholls, Loetscher & Rademacher [37] found no correlation between rightward deviations of a soccer ball kick and the spatial bias elicited when pointing towards the goal midpoint, and Hatin et al. [21] observed no relationship between doorway collision frequency and bias on the standard line bisection task, with more *left*-sided collisions identified overall. Importantly, the majority of these studies recruited a young participant cohort, and age-related effects have rarely been investigated in naturalistic tasks. Hatin et al. [21] found that, although lab-based measures tend to show a consistent rightward shift of spatial bias in older age, there was no difference in left versus right doorway collision frequency between young and older adults, and further similarities between age groups have been observed in American driving collision statistics [38]. These studies indicate a need for further research into naturalistic spatial biases in the older population and how they might relate to laboratory measures of spatial attention asymmetries.

In this study we were interested in assessing how a more complex behaviour (lane-keeping during simulated driving) may be associated with spatial attention asymmetries, and in particular whether a rightward shift in older age is also observable on this task. The ability to quickly and accurately process, and act upon, spatial information is an essential component of safe driving in young and older adults alike. Research surrounding the use of in-car automatic lane departure warning software has identified that lane-departure warnings are generally activated more often in older adults than in young [39]. However, spatial asymmetries are difficult to study in driving behaviour due to the potential confound of learned behaviour associated with left- or right-lane driving experience. For example, Dutch right-lane drivers naturally adopt a lateral lane position that is slightly to the right of centre, probably as a learned behaviour to avoid oncoming traffic approaching from the left, or to overcompensate for being asymmetrically seated on the left side of the vehicle [40,41]. Similarly, right-lane drivers in South Korea adopt a right-of-centre position when instructed to drive at the lateral midpoint of a single-carriageway road [42], and the majority of unintentional lane-drift crashes on straight roads in a sample of 5,470 road traffic accidents in the Unites States were towards the right hand side [43]. This appears to be mirrored in the left-driving population: Lenné, Triggs & Redman [44] observed a drift towards the left edge of the road over time in Australian drivers, and in Scotland, 60.05% of injury-causing accidents involving lane departures are towards the left-side of the carriageway, with right-sided departures less frequent (39.95%: [45]).

Interestingly though, there is evidence of cross-cultural behavioural similarities that may point towards the influence of spatial attention biases during driving. Friedrich, Elias & Hunter [38] found a similar pattern of left-sided vehicle collisions as observed in the Scottish traffic records, in the right-lane driving American population. This was contrary to expectations and importantly, they found no difference in the laterality of collision behaviour between young and older adults. However, Hämäläinen et al. [46] recently found that older, but not younger, right-lane driving Finnish adults tended to erroneously cross the lane boundary to the left during simulated driving, whilst performing a simultaneous, high-load perception task. The older adults also had a rightward perceptual bias, indicated by more erroneous rightward responses during bilateral visual target presentation, suggesting that their leftward driving error may be related to this rightward perceptual bias. Further evidence of a leftward perceptual biases during driving was gained from Benedetto et al. [47] who identified a left-sided attentional preference in Italian right-lane drivers using eye-tracking during a simulated Lane Change Task [48]. In the Lane Change Task, participants are instructed to move their vehicle into one of three lanes, in response to instructions presented on bilaterally-situated signs on the left and right sides of the track. Participants were found to look towards the left-situated signs more frequently than those placed on the right, even though road signs are normally right-situated on Italian roads. In summary, some aspects of driving behaviour are likely to be influenced by learned experience, but natural asymmetries of lateral spatial processing, such as pseudoneglect, may also be involved.

We aimed here to investigate the relationship between two standard, lab-based measures of spatial attention bias (the LANDMARK and LINE BISECTION tasks) and a simulated LANE KEEPING task, in young and older adults with prior experience of left-lane driving. We modified the Lane Change Task used in Benedetto et al. [47] so that the instructions were similar to the standard line bisection task (i.e. find and maintain the midpoint of the lane). By testing a left-lane driving population, we aimed to de-couple the predicted effects of prior driving experience from spatial attention asymmetries. Specifically, we predicted that if lane positioning is influenced by prior driving experience, both young and older adults would naturally adopt a left-of-centre driving position mirroring the rightward preference of European drivers. Conversely, they would position themselves to the right-of-centre if driving position is related to a spatial attention asymmetry (i.e. pseudoneglect), and we would also expect to observe an inverse correlation between lateral driving position and the landmark and line bisection tasks. We predicted that older adults would lose their group-level leftward bias on the landmark and bisection tasks, and expected their lane-keeping to be less lateralised than the younger group as a result.

## 2. Method

### 2.1. Participants

78 right-handed adults were recruited. One young participant was excluded from statistical analysis due to a landmark task bias score of >2.5 standard deviations above the group mean (PSE = 28.91). The remaining 77 participants comprised 38 young adults (27 females, mean age = 21.53, range = 18–34, SD = 4.35) and 39 older adults (19 females, mean age = 70.38, range = 60–86, SD = 6.03). All had normal or corrected-to-normal vision with glasses or contact lenses and held a valid UK driving licence. Young adults had on average 3.08 (SD = 3.42) years of driving experience since gaining their licence, and the older group 47.4 (SD = 10.16) years. The study was approved by the University of Glasgow College of Science and Engineering ethics committee and written, informed consent was obtained from each participant.

### 2.2. Procedure

Participants were asked to indicate their subjective alertness on a linear scale (0 = almost asleep, 100 = fully alert) before and after testing. Young adults were tested in one single experimental session, with each participant completing 3 tasks (landmark, line bisection, and lane-keeping) in a counterbalanced order across participants. The older adults completed the 3 tasks as part of a larger experiment involving a total of 6 counterbalanced spatial attention tasks: landmark, line bisection, greyscales, grating scales, lateralised visual detection (as per [7]), with the addition of the lane-keeping task. All 6 tasks were undertaken twice: once per session, across 2 days separated by a minimum of 24 hours. The data reported here was obtained during the first of the two testing sessions.

### 2.3. Landmark and line bisection tasks

In the young group, the landmark and line bisection tasks were performed on a Dell Precision T3400 PC with a CTX Ultra 18” monitor (1280x1024 pixel resolution and 85Hz refresh rate). Older adults were tested on a Dell Precision 380 PC with a 19” Dell 1908FP Ultra Sharp LCD flat screen monitor, with a 1280x1024 pixel resolution. Viewing distance was kept constant at 0.6m with a chin rest.

#### 2.3.1. The landmark task

**Fig 1:**
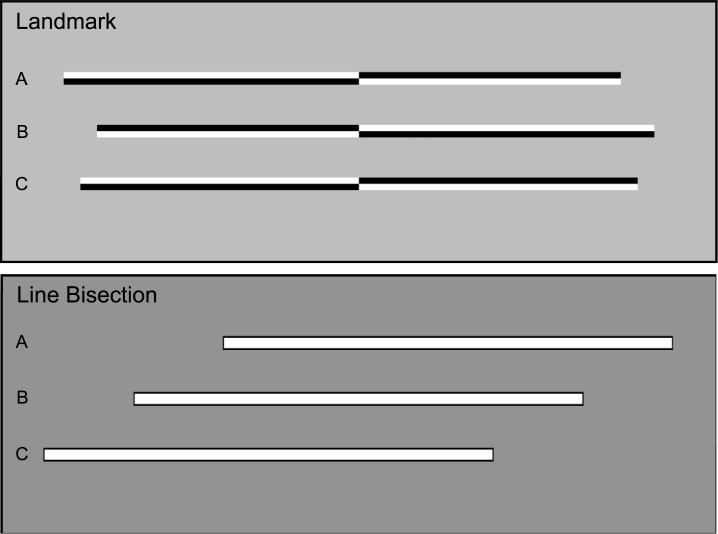
The landmark and line bisection task stimuli. *Landmark task*: A) The right side is shorter than the left by 48 pixels, B) The left side is shorter than the right by 48 pixels and C) Both sides are of equal length. *Line bisection task*: A) Line jittered 160 pixels to the right of centre, B) Centred, and C) Jittered 160 pixels to the left of centre.

*Stimuli*: Stimuli were identical to those used in Learmonth et al. [7] and were originally adapted from [49] and [2]. Horizontal lines of 100% Michelson contrast were presented individually on a grey background (luminance = 179, hue = 160) at the vertical midpoint of the screen. Each line measured 800x14 pixels (approximately 22.4x0.4cm, 21.15x0.33° visual angle. Each line was transected vertically at the centre of the screen but the length of the left and right sides of the line varied across trials to enable psychometric curve fitting. A total of 17 different stimuli, varying in their asymmetry, were used: 1 where the left and right sides were of equal length, and a series of stimuli where the left (or right) side was either 6, 12, 18, 24, 30, 36, 42 or 48 pixels shorter (or longer) than the left. One landmark task block consisted of 136 trials (8 repetitions of 17 stimuli: 4 repetitions where the upper left/lower right were shaded black and 4 repetitions where the these quadrants were shaded white, as per Fig 1).

*Procedure*: In each trial, a centred fixation cross appeared for 1000ms, followed by one of the landmark stimuli for 150ms. The fixation cross then reappeared until a two-alternative forced-choice key press was made. Participants were instructed to press “v” with their right index finger if they perceived the left side of the line to be shorter than the right, and “b” with their right middle finger if they perceived the right side of the line to be shorter than the left.

*Analysis*: The percentage of trials in which the participant judged the left to be shorter was calculated separately for each of the 17 stimuli. Psychometric functions were fitted to this data, separately per subject, using a cumulative logistic function:

$$f(\mu ,x,s)=1/(1+\exp\;\left(\frac{x-\mu }{s}\right))$$

where *μ* is the point on the x-axis that corresponds to 50% left and 50% right-response rate, *x* represents the transector locations and *s* is the psychometric curve width. The point of subjective equality (PSE) and curve widths were extracted. The PSE is a measure of the subjective horizontal midpoint of the landmark line and is used to quantify spatial attention bias, whereas the curve width estimates the overall precision of these judgements. A wide (large) curve width value indicates low precision and a narrow (small) curve width value high precision.

#### 2.3.2. The line bisection task

*Stimuli*: Individual horizontal white lines were presented on a grey background at the vertical midpoint of the screen (Fig 1). Each line measured 805x15 pixels (approximately 22.4x0.4cm, 21.15x0.33° visual angle). The outermost two pixels bordering the line were shaded black. The line was jittered along the horizontal axis on a trial-by-trial basis at 9 different locations (0 = centred on the screen, and 40, 80, 120 and 160 pixels to the left and right of centre (approximately 0.95, 1.9, 2.85 & 3.8°). The mouse pointer appeared at the upper horizontal midpoint of the screen at the start of each trial (screen co-ordinates: X = 640, Y = 40 pixels; 11.17° above the fixation cross). One line bisection task block consisted of 108 trials (12 repetitions of 9 stimulus locations).

*Procedure*: In each trial, a fixation cross appeared for 1000ms, then one of the 9 stimuli appeared until either a response was made, or a maximum of 6 seconds had passed without a response. The next trial appeared thereafter, with the mouse pointer reset at the start position. Participants were instructed to move the mouse down towards the line using their right hand, and to use the left mouse button to click on the horizontal midpoint of the line as accurately as possible.

*Analysis*: The horizontal and vertical co-ordinates of the mouse click location were logged in E-Prime. The subjective horizontal midpoint of the line (i.e. the clicked x-coordinate) was subtracted from the true midpoint location to generate a spatial bias score for each trial, in pixels relative to midpoint. Trials that were more than 2.5 SD above and below the individual’s mean bias score were excluded, the majority of which appeared to be due to mouse clicks made in error when lowering the mouse towards the line (Young: 43 trials = 1.05% of total, Older: 83 trials = 1.97%). An overall mean line bisection bias was then calculated per individual.

#### 2.3.3. The lane-keeping task

**Fig 2:**
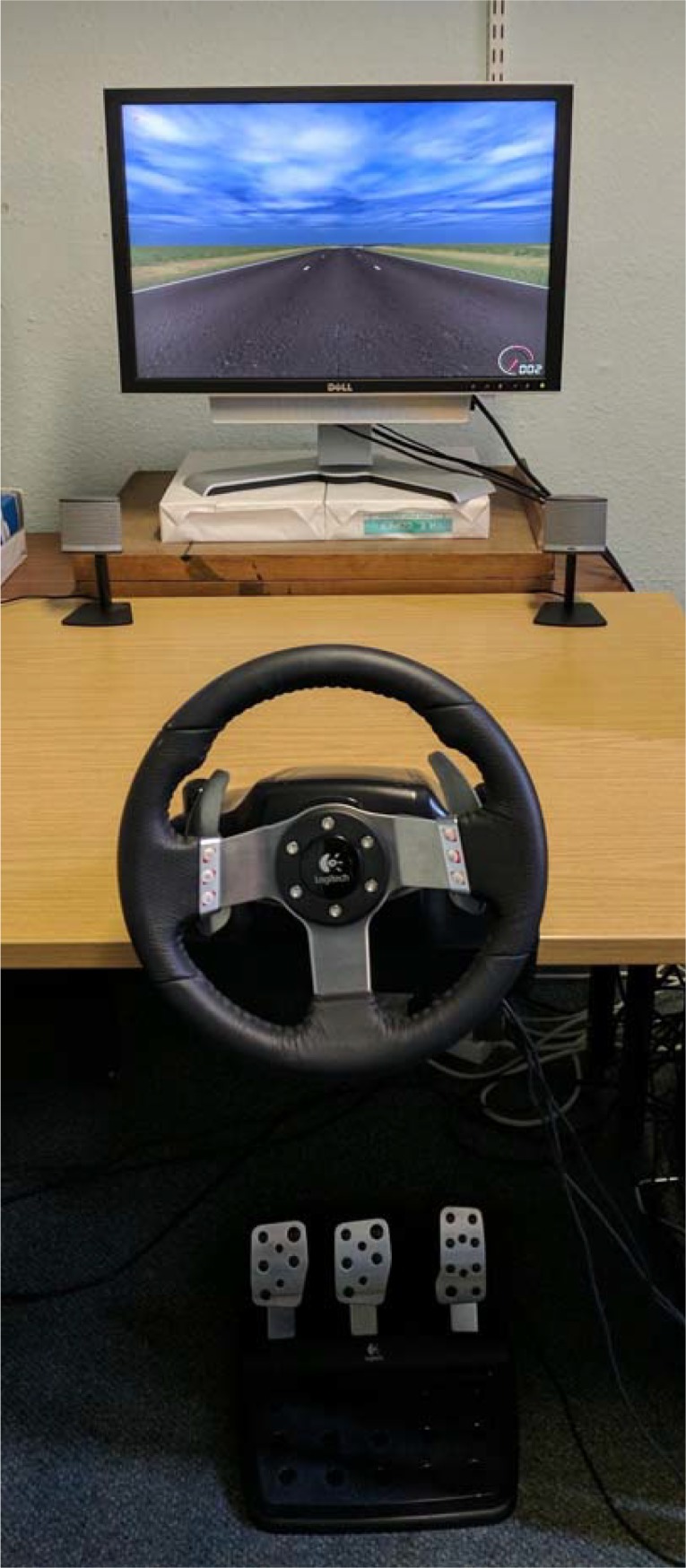
The lane-keeping task experimental setup.

The lane-keeping task was adapted from the Lane Change Task, an ISO-standardised driving simulation software that is commonly used to assess the effects of secondary tasks on driving performance within the laboratory setting (ISO 17387:2008 [48]). In both young and older groups, the task was presented on a Dell Precision T7400 PC with a Dell Ultrasharp 2408WFP 24” LCD computer screen (1680x1050 pixel resolution). Participants were seated approximately 1m directly in front of the screen, with a Logitech G27 gaming steering wheel attached to the table (Fig 2). Three pedals were situated on the floor, representing the clutch (left pedal), brake (middle) and accelerator (right), as per a standard manual transmission vehicle. The position of the chair and pedals were adjusted to comfortably accommodate each participant. The Lane Change Task software produced a simulated engine noise when the accelerator pedal was pressed, but no other vehicle details (vehicle frame, windshield, gearstick etc.) were present.

*Procedure*: The instructions for the Lane Change Task were modified to enable a direct comparison between the 3 tasks. Participants were instructed to keep both hands on the steering wheel and, using the right foot pedal to accelerate, to maintain a constant speed of 60mph (the maximum possible speed, as indicated by a speedometer at the lower right of the screen) along the straight sections of the track. The course began with a short straight section, followed immediately by a right-hand bend. The track then straightened, and participants were instructed to accelerate to 60mph and position themselves at the middle of the centre lane by the time they reached the yellow “start” flag. They were then instructed to maintain a constant position at the centre of the middle lane for the duration of the straight section (Lap 1), using the steering wheel to adjust if necessary. Each lap involved a 3km long straight track with no other cars or pedestrians present, taking approximately 3 minutes to complete at a constant maximum speed of 60mph, and the simulated width of each of the 3 lanes was 3.85m wide (Fig 2). In the standard LCT task, participants are usually instructed to change lanes in response to instructions presented on 18 signs that are simulated at 150m intervals, on the left and right edges of the track. Here they were specifically instructed to ignore these signs. After they had driven past the final flag of Lap 1, the second lap was approached via a right-hand bend, and the third lap via a left-hand bend. Each participant completed 3 laps of the track, with the first lap considered a practice, to familiarise themselves with the simulator controls and procedure.

*Analysis*: The horizontal position of the car within the middle lane (deviation from the midpoint in metres) was logged by the LCT software throughout the experiment, where 0 = the veridical midpoint of the lane, a negative value representing left-of-centre lane position and positive value a right-of-centre position. The position of the car as it passed the start flag plus each of the 18 instruction signs was extracted for each participant. The mean lane position, in metres, was then calculated separately for each of the 2 test laps, per person, and finally, the grand average of the two laps was calculated.

## 3. Results

The full dataset for this study is available at https://osf.io/53c7w/.

### 3.1. Subjective alertness

A 2x2 ANOVA (time: pre vs post experiment x age: young vs older) identified an overall decrease in alertness over time, [F(1,74) = 8.11, p = 0.006, ηp^2^ = 0.1], and generally higher alertness ratings in the older group [F(1,74) = 23.74, p<0.001, ηp^2^ = 0.24]. Neither group experienced a larger alertness reduction pre- vs post-test than the other (time x age interaction: F(1,74) = 0.33, p = 0.57, ηp^2^ = 0.004, mean alertness scores: young pre-test = 66.71, post-test = 61.79; older pre-test = 84.49, post-test = 77.69).

### 3.2. Mean spatial biases

**Fig 3:**
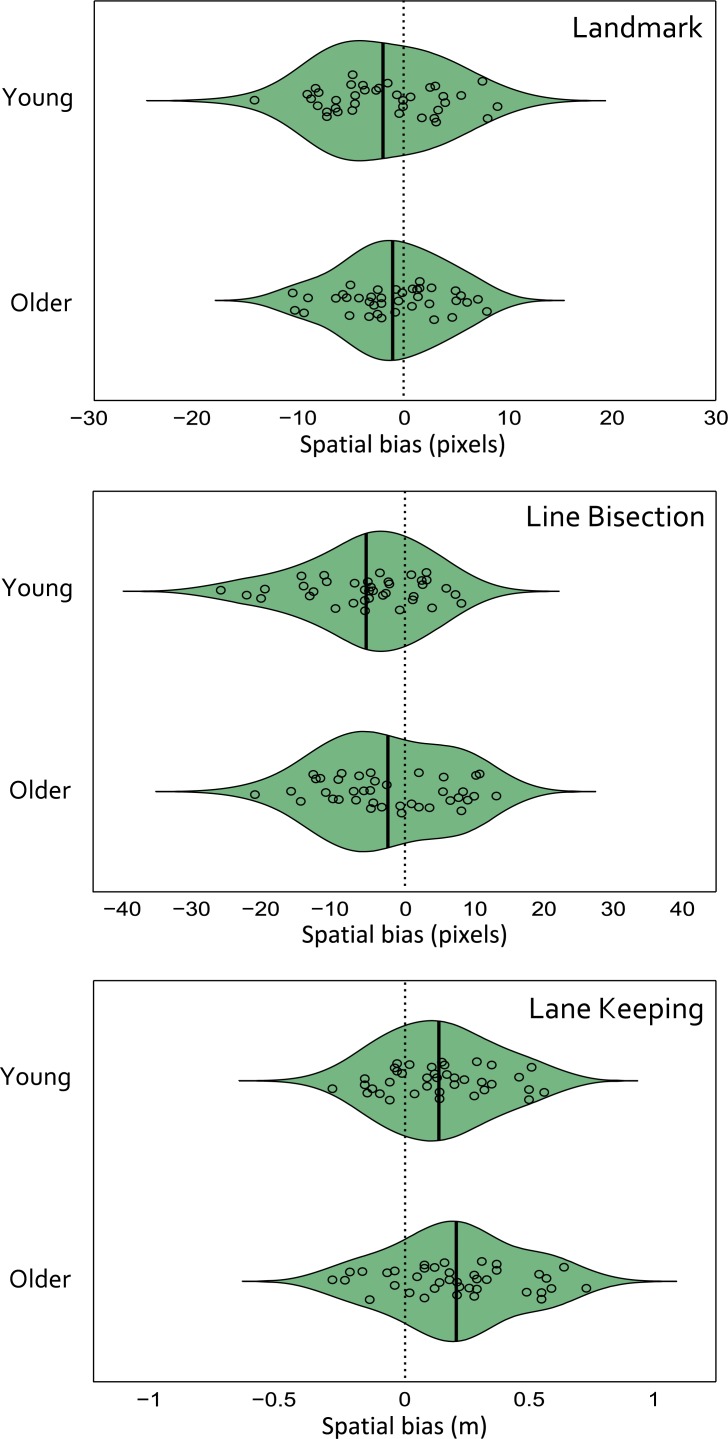
Mean spatial biases for the landmark (in pixels), line bisection (pixels) and lane-keeping tasks (metres). Individual biases are overlaid.

Young adults had a leftward spatial attention bias on the landmark task (mean PSE = -2.12 pixels, one-sample t-test against zero (i.e. no bias) t(37) = 2.35, p = 0.024) and on the line bisection task (mean bias = -5.53 pixels, t(37) = 4.04, p<0.001), indicative of pseudoneglect in both tasks. Although the older adults had a mean leftward bias for both the landmark and line bisection tasks, these were not significantly different from zero (landmark task mean PSE = -1.19 pixels, t(39) = 1.53, p = 0.13; line bisection task mean bias = -2.39 pixels, t(39) = 1.7, p = 0.098) (Fig 3).

In the Lane-Keeping Task, the mean lateral positions in Lap 1 and Lap 2 were strongly correlated in both age groups (Lap 1 vs Lap 2: young r(38) = 0.78, p<0.001, older r(39) = 0.82, p<0.001). Therefore, the grand average lateral lane positions (mean of Laps 1 & 2) were used in all subsequent analysis. Mean spatial bias was to the right of centre for the young adults (mean position = 0.14m, one-sample t-test against zero (i.e. no bias) t(37) = 3.95, p<0.001) and also to the right of centre for older adults (mean position = 0.21m, t(38) = 5.05, p<0.001) (Fig 3). Independent samples t-tests were then used to compare the spatial biases between young and older adults, separately across each of the 3 tasks, but these identified no age-related differences for any task (landmark: t(75) = 0.79, p = 0.44, line bisection: t(75) = 1.6, p = 0.12, lane position task: t(75) = 1.31, p = 0.2).

**Fig 4:**
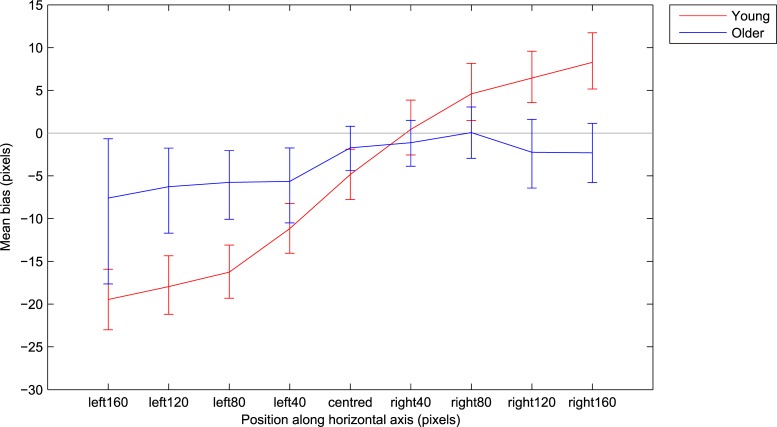
Mean spatial biases obtained for lines presented at each location along the horizontal axis, separately for each age group, with 95% confidence intervals (2000 bootstrapped samples).

The line bisection task was further analysed to probe for age-related differences at each of the 9 line positions along the horizontal axis. A 9x2 mixed ANOVA (position x age) identified a main effect of line position (F(8,600) = 43.85, p<0.001, ηp^2^ = 0.37) where lines positioned to the left of the screen were generally bisected further to the left than lines positioned to the right of the screen. There was no main effect of age (F(1,75) = 0.77, p = 0.38, ηp^2^ = 0.01) but there was an age x position interaction (F(8,600) = 18.31, p<0.001, ηp^2^ = 0.2). 95% confidence intervals were bootstrapped (2000 samples) for each position, per age group, and these are shown in Fig 4. Generally, young adults were more susceptible to the lateral position of the line, with the left-positioned lines bisected further to the left in young adults compared to the older adults (non-overlapping 95% CIs when the line was placed 80 and 120 pixels to the left of centre), and further to the right when lines were positioned towards the right of the screen (non-overlapping 95% CIs when the line was placed at 120 and 160 pixels to the right of centre).

### 3.3. Inter-task correlations

**Fig 5:**
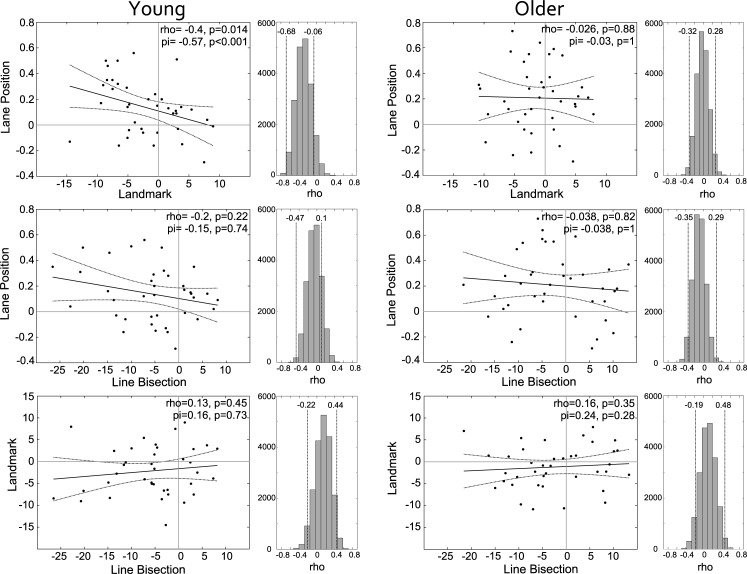
Correlation plots showing the relationship between the lane position, landmark and line bisection tasks for young and older adults, with regression line and 95% confidence intervals. Histograms show the distribution of 20,000 bootstrapped samples of the Spearman’s rho correlations, with 95% confidence intervals.

The 3 tasks were then inter-correlated using Spearman’s rho and Shepherd’s pi ([50]: a robust correlation method which adjusts for the influence of outlying data points), separately for young and older adults (Fig 5).

*Young adults*: The lane position and landmark tasks were moderately negatively correlated: rho(38) = -0.4, p = 0.014 (significant at Bonferroni corrected α = 0.017); Shepherd’s pi: r(38) = -0.57, p<0.001; bootstrapped 95% confidence intervals (CI) after 20,000 iterations = [-0.68, -0.06] with a leftward spatial bias on the landmark task associated with a mean rightward lane position. The lane position and line bisection tasks failed to correlate rho(38) = -0.2, p = 0.22; pi = -0.15, p = 0.74; CI = [-0.47, 0.1] and the landmark and line bisection tasks also failed to correlate, confirming a lack of inter-task relationship between these 2 tasks as per Learmonth et al. [7]: rho(38) = 0.13, p = 0.45; pi = 0.16, p = 0.73; CI = [-0.22, 0.44].

*Older adults*: None of the 3 tasks were correlated in the older adults: *lane position vs landmark*: rho(39) = -0.026, p = 0.88; pi = -0.03, p = 1; CI = [-0.32, 0.28], *lane position vs line bisection*: rho(39) = -0.038, p = 0.82; pi = -0.038, p = 1; CI = [-0.35, 0.29], *landmark vs line bisection*: rho(39) = 0.16, p = 0.35; pi = 0.24, p = 0.28; CI = [-0.19, 0.48].

## 4. Discussion

The aim of this study was first of all to examine whether spatial biases can be elicited during a simulated driving task, and secondly whether these biases change with age, similar to standard lab-based measures. As we, and others, have previously reported, young adults exhibited a group-level leftward pseudoneglect bias on both the LANDMARK and LINE BISECTION tasks, whereas older adults had no group-level bias to either side of space. Both young and older groups positioned themselves significantly to the right of centre in the LANE KEEPING task, however there were no age-related differences in any of the 3 tasks when the groups were compared directly. Importantly, although biases failed to correlate across the 3 tasks in older adults, lateral lane position was moderately negatively correlated with the landmark task in the younger group (rho = -0.4, pi = -0.57).

### 4.1. Spatial bias and lane keeping in young adults

In finding no correlation between the landmark and line bisection tasks in young adults, we have replicated our previous results in Learmonth et al. [7], where we found no significant inter-task correlation between these two perceptual tasks. Here we have extended this work to demonstrate a similar lack of relationship between these tasks in older adults. We again conclude that although each of these tasks may elicit significant group-level spatial biases individually, the unique cognitive demands of each task renders them non-interchangeable in spatial attention research. In light of this, the moderate correlation observed between the lane keeping and landmark tasks in young adults is notable, particularly given that prior evidence of correlations between line bisection and naturalistic measures has been mixed [21,24,35–37].

The distinction between lateralised perceptual and behavioural biases is central to the interpretation of these results. Our findings are in broad agreement with much of the previous literature on spatial attention asymmetries, which has described a perceptual over-estimation of the left side of space, concurrent with a compensatory behavioural bias towards the right during more naturalistic tasks, such as doorway navigation [24,36,37]. Similar to observations in lab-based studies of spatial asymmetries, perceptual biases towards the left side of space have been demonstrated during driving in right-lane driving populations [47]. This observation is interpreted as evidence of a universal perceptual bias towards the left side of space (*pseudoneglect*), given that these participants all had prior experience of road signs situated on their right side. If we are correct in supposing that *behavioural* biases arise in direct response to leftward *perceptual* biases (specifically, where the left side of space is perceived to be larger than the right), then we would expect all drivers to position themselves to the right of centre, regardless of whether they are experienced in driving on the left or the right side of the road. However, if lane position is purely influenced by prior driving experience, our left-lane driving participants should have naturally positioned themselves to the left of centre, given their experience of avoiding oncoming traffic approaching to their right, and mirroring findings in right-lane driving populations [40–42]. Our observation here that both young and older adults adopted a significant mean *rightward* lane position leads us to conclude that at least some aspects of driving behaviour are likely to be cross-cultural and influenced by spatial attention asymmetries rather than learned strategies. Indeed, the moderate negative correlation observed in young adults between the lane keeping task and the psychophysical landmark task supports the interpretation that both tasks reflect a stable property of the cerebral attention networks, contrary to our results in Learmonth et al. [7]. It is therefore likely that the left side of space was perceptually over-estimated and participants situated themselves towards the right of centre as a form of behavioural spatial compensation. Given that prior research has focused on groups of *either* right- or left-lane drivers, it would now be of interest to perform a direct, cross-cultural test of perceptual and behavioural biases during driving to disentangle the effects of natural spatial attention asymmetries from learned behaviour.

### 4.2. Spatial bias and lane keeping in older adults

Although our older adults failed to show any lateralised biases on the landmark and line bisection tasks, similar to many previous findings [9–20], there were no significant age-related differences when each task was probed using independent samples t-tests. We had initially predicted that, if lane-keeping behaviour is determined by perceptual attention asymmetries, our older adults would prove less lateralised than the younger group on all tasks. However, the lack of significant difference between the two age groups in the landmark and line bisection tasks makes this hypothesis difficult to answer conclusively here. Nevertheless, the mean bias reduction observed here in the older group does generally agree with previous studies. What is certainly contrary to our initial predictions is that older adults, like the younger participants, had a significant *rightward* positioning on the lane keeping task. One possible explanation for this may be that lateralised spatial asymmetries are elicited more effectively when older adults are engaged in more complex and ‘real-world’ behavioural tasks, and that abstract lab-based and/or psychophysical measures of perception may fail to capture subtle spatial attention asymmetries that are, in fact, still present in this group.

It is unlikely that the extensive prior driving experience in the older group gave rise to this maintained rightward position. Our older participants were all highly experienced left-lane drivers, with a mean of 47.4 years of experience, versus 3.08 years in the young group. We would therefore have expected to observe either a position closer to the true centre, in response to perceptual learning, or a higher incidence of left-of-centre positioning due to more experience of actively avoiding traffic approaching from the right. Given that our results showed a positioning towards the opposite side, it is unlikely that this prior experience substantially influenced behaviour on the lane position task. It is also important to note that we observed a considerable number of older adults who, individually, did maintain a leftward bias in the landmark and line bisection tasks similar to the young group (see [9,10,22]). These older adults may represent a highly-functioning subset of the population, given that older participants are often self-selective in volunteering for lab-based experiments, and full-profile cognitive testing was unfortunately not undertaken here. This could also be an important factor in other studies which have reported varying findings, as the cognitive and physical health status of the older group is rarely examined or reported and may not be fully representative of typical aging.

### 4.3. Limitations

It is also possible that the different viewing distances used in the perceptual tasks and the driving task could have influenced the results obtained in this study. This is particularly relevant given that the driving task was not an entirely naturalistic representation of real-world driving behaviour, but was instead very similar in design to the perceptual line bisection task. In the line bisection task, participants were instructed to mark the midpoint of a horizontal line. In the lane keeping task they had to identify the lane midpoint and maintain this position, adjusting if necessary. The driving task was completed using a standard desktop computer, thereby removing the contextual cues that may be associated with natural driving behaviour. It is therefore important to question whether this experimental design was naturalistic enough to elicit a rightward behavioural bias, or whether the task instead represents a perceptual lane bisection task similar to the landmark and line bisection. In perceptual spatial attention tasks, leftward pseudoneglect biases are typically observed within peri-personal space, with a rightward shift of perception often identified when the perceptual judgement takes place in extra-personal space [51–55]. Here, the landmark and line bisection tasks were performed within a peri-personal viewing distance of 0.6m, whereas participants were 1m from the computer screen during the lane keeping task, and thus in extra-personal space. We could, therefore, expect a perceptual bisection task, undertaken in extra-personal space, to result in a significant rightward bias, as observed here. However, we have previously demonstrated leftward perceptual biases in young adults using a ≥1m viewing distance [56,57] and therefore consider this explanation to be unlikely.

As a final consideration, it remains unclear how these results can be integrated with the disproportionate number of collisions on the left side of vehicles in right-lane driving populations [38]. Right-lane drivers would be expected to collide more frequently on the right if vehicle collisions arise purely as a result of a rightward behavioural compensation for a leftward perceptual asymmetries. We would also expect slightly more frequent right-sided collisions in American drivers if they actively avoided oncoming vehicles approaching to their left. This explanation would also align with Thomas et al. [23] who found more frequent right-of-centre doorway deviations in Swiss adults (right-lane drivers), with a reduced bias in Australians (left-lane drivers), which was attributed to differences in prior driving experience. However, it is possible that the higher frequency of left collisions in Friedrich et al. [38] was a result of traffic hazards (i.e. oncoming vehicles) approaching the left side more often than the right, rather than a result of compensatory (either perceptual or avoidant) lateral lane positioning. Similarly, the higher frequency of left-sided lane departures in the Scottish traffic records could be attributed to avoidant actions towards the left, or forced departures as a result of right-sided collisions. Unfortunately the reasons for these accidents are not specified, but would provide a clearer picture of the role of other vehicles in lateralised aspects of driving behaviour. Given that these vehicle collision statistics are drawn from thousands of reported driving accidents, and are therefore highly naturalistic behaviours by definition, we must remain mindful that driving is a highly complex process involving an interplay of multiple variables (e.g. road width, bends, speed limits, single carriageway vs motorway, the presence of other vehicles and pedestrians, congestion levels and standard manoeuvres such as turning corners) in addition to the possible contribution of lateral attention biases. As mentioned previously, although the lane keeping task we developed involved a more ecologically valid test of spatial attention compared to many psychophysical measures, our driving simulator setup was admittedly not completely naturalistic. In a natural left-lane driving scenario, the driving seat is positioned asymmetrically on the right side of the car, whereas our setup here involved a single chair positioned directly in front of the computer screen. Our participants may have overcompensated for this unfamiliar positioning by placing themselves slightly too far to the right of centre when asked to maintain a central position on the simulated track. Yet, this would still not explain the presence of a correlation between the driving and the landmark task in young adults. Secondly, we required participants to maintain a fixed mid-lane position in the middle of three lanes for an extended period of time, and this behaviour is rarely—if ever—required during normal day-to-day driving. Maintaining an appropriate position within a lane usually only requires that the vehicle is positioned broadly within the specified lane (a space that is almost always wider than the car), and not necessarily fixed at the midpoint. It would be of value now to improve the ecological validity of this task by assessing whether lab-based spatial attention tasks are correlated with lane positioning during regular carriageway driving.

In conclusion, we have demonstrated that both young and older British adults adopt a significantly right-of-centre position in a simulated lane-keeping task, contrary to predictions in a left-lane experienced population. There was a moderate negative correlation in young adults between the computerised landmark task and the lane position task, indicating that lateralised asymmetries of visuospatial perception likely impact on driving behaviour. These results suggest that the development of a deeper understanding of natural lateralised biases of visual attention, and how they relate to driving behaviour, is important for future research into the development of driving safety measures, particularly into older age.
